# From the Classical Schmid Factor to Tensor-Based Global Measures: A Critical Review and Practical Advice for Beginners

**DOI:** 10.3390/ma19173803

**Published:** 2026-09-07

**Authors:** Dabiao Xia, Manoj Gupta, Long Xu

**Affiliations:** 1 Anhui Engineering Research Center for Agricultural Product Quality Safety Digital Intelligence, Fuyang Normal University, Fuyang 236037, China; 2 College of Design and Engineering, National University of Singapore, Singapore 117575, Singapore; mpegm@nus.edu.sg; 3 Jihua Laboratory, Foshan 528000, China

**Keywords:** Schmid law, global Schmid factor, magnesium alloys, deformation twinning, stress tensor, critical resolved shear stress

## Abstract

The Schmid factor provides a compact link between an applied load and the resolved shear stress on a selected crystallographic system. For uniaxial loading, m=cosφcosλ, and its maximum magnitude is 0.5. This familiar result is conditional on the uniaxial stress model rather than a universal bound. Under a symmetric Cauchy stress tensor σ, the corresponding projection is $\tau_{RSS}=b^{T}\sigma n$. A dimensionless global Schmid factor (GSF) further requires a stated reference stress. When the von Mises equivalent stress $\sigma_{eq}=\sqrt{\frac{3}{2}s:s}$ is used, with orthogonal unit vectors b and n and $\sigma_{eq}>0$, $|GSF|\leq \frac{1}{\sqrt{3}}$, the uniaxial limit is recovered as a special case. This review separates the tensor projection from effective or modified factors defined for particular loading conditions. SF and GSF describe the favorability of a crystallographic system under a prescribed stress, but they are neither intrinsic material properties nor complete activation criteria. Magnesium alloys are used to examine slip-system competition, twinning polarity, texture, local stress redistribution, and differences in critical resolved shear stress. In addition to calculation procedures, limiting cases, reporting requirements, and research directions, common learning difficulties and a progressive route for interpreting Schmid-based measures are discussed.

## 1. Introduction

Relating an external load to a crystallographic deformation mechanism is a central multiscale problem in materials science. Macroscopic yielding is measured at the specimen scale, whereas plastic flow proceeds through dislocation motion or deformation twinning on specific crystallographic systems. Schmid’s law provides the basic connection: the applied stress is projected onto a selected plane and direction, and the resulting resolved shear stress is compared with the critical resolved shear stress (CRSS) [1,2].

The classical treatment considers a single crystal under uniaxial tension or compression and gives m=cosφcosλ, where φ is the angle between the loading axis and plane normal, and λ is the angle between the loading axis and slip direction [3]. Its simplicity makes it useful for orientation analysis, but it also encourages the upper bound of 0.5 to be separated from the assumptions used in its derivation. This becomes problematic when EBSD software requires a sample-frame stress tensor, or when signed factors and values above 0.5 are reported for non-uniaxial loading.

The distinction is relevant because many processing and service conditions are multiaxial. Rolling, extrusion, forging, contact loading, asymmetric rolling, severe plastic deformation, and residual-stress fields involve combinations of normal and shear components. In addition, the stress carried by an individual grain can deviate from the macroscopic stress because of elastic–plastic anisotropy and grain-neighbor constraints [4,5,6,7,8,9]. These effects are pronounced in magnesium alloys, where basal, prismatic, and pyramidal slip compete with polar twinning, and mechanism selection depends on stress sign, texture, CRSS ratios, temperature, strain path, and local constraint [10,11,12,13,14,15].

Several studies have, consequently, introduced effective, global, or modified Schmid factors for non-uniaxial loading. Chen et al. incorporated normal and shear stresses when analyzing slip and twin-variant selection in Mg-3Al-1Zn alloy [16]. Xia et al. evaluated the influence of stress state using von Mises normalization [17], while Wang et al. extended the calculation to three-dimensional stress states and retained the directional information associated with shear components in the original coordinate system [18]. Although related, these formulations do not necessarily use the same numerator, denominator, or physical assumptions [19].

Against this background, the present review places the classical Schmid factor, tensor-based resolved shear stress, von Mises-normalized GSF, and selected effective or modified formulations within a common framework. Particular attention is given to definitions, bounds, sign conventions, coordinate transformations, and the distinction between a geometric projection and a constitutive activation criterion. The discussion is also intended to support first-time users, for whom errors in stress normalization or physical interpretation are often more consequential than errors in the algebra itself.

This dual emphasis is useful because the same misconceptions appear in both classroom exercises and research post-processing. A factor of 0.5 is sometimes treated as a universal limit, the largest factor is taken as proof of activation, and an EBSD Schmid map is interpreted as a local-stress measurement. Correcting these points requires a clear physical picture of what is being projected, followed by explicit checks on the stress state, resistance, and experimental evidence.

## 2. The Classical Schmid Factor and What It Actually Says

### 2.1. Uniaxial Loading

**Figure 1:**
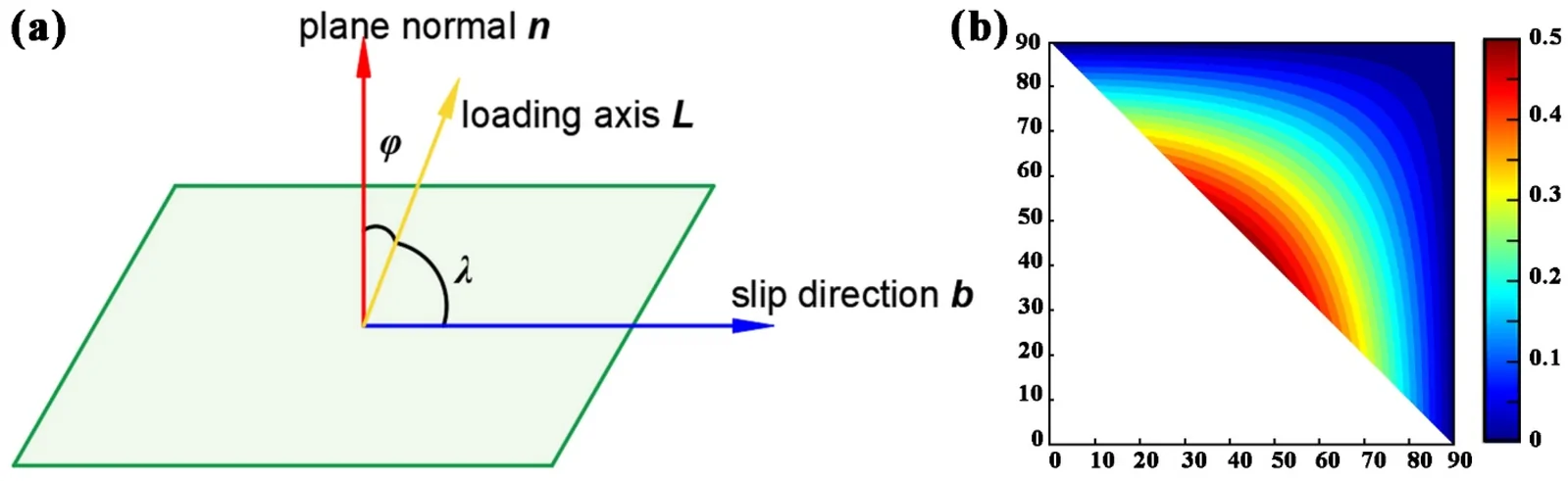
(**a**) Classical uniaxial Schmid geometry and (**b**) the conditional origin of |m|≤0.5. X and Y axis mean λ and φ in degree.

Figure 1a shows a schematic diagram of the classical Schmid factor. Let L be a unit vector in a uniaxial loading direction, b a unit vector in the slip direction, and n a unit normal to the slip plane, with b·n=0. For an applied uniaxial stress σ, the resolved shear stress on the selected system is

(1) $$\tau_{RSS}=\sigma \left(L\cdot b\right)\left(L\cdot n\right)=\sigma cos\lambda cos\varphi$$

The classical Schmid factor is, consequently,

(2) m=cosφcosλ

For ordinary dislocation slip, the directions b and −b are usually treated as equivalent, and |m| is reported. Twinning is polar, and the shear sense must, therefore, be retained when distinguishing an admissible variant from one requiring the opposite shear [10,11]. The sign convention should be stated whenever slip and twinning systems are compared.

From the geometric relationship, it can be seen that φ and λ are coupled, and Figure 1b shows the correlation between the values of the two angles. The maximum magnitude under uniaxial loading is 0.5. It occurs when the loading axis is equally inclined to b and n, i.e., $\left|\lambda \right|=\left|\varphi \right|=45{}^{\circ}$ for the favorable sign convention. The bound follows from the specific uniaxial stress form σ=1 together with b·n=0; it should not be attributed to a generic “rank-one form” without writing that stress tensor explicitly. The sign of m must also be interpreted consistently with the chosen tension/compression and slip/twin direction conventions.

### 2.2. From a Loading Axis to a Stress Tensor

Under a general stress state σ, there may be no unique loading direction. The traction acting on a plane with normal n is τ=σn, and the component of that traction along b is

(3) $$\tau_{RSS}=b^{T}\sigma n$$

Equation (3) is the durable statement of Schmid projection. Equation (1) is recovered when σ happens to be uniaxial. This change of viewpoint is modest mathematically but substantial conceptually: the applied stress is not first reduced to an imagined force direction. Instead, the full tensor acts on the plane normal, and the resulting traction is projected onto the deformation direction [20,21].

The hydrostatic part makes no direct contribution to the Schmid resolved shear stress. A convention-safe decomposition is $\sigma =S+\sigma_{m}I$, where $\sigma_{m}=tr(\sigma )/3$, and S is deviatoric. Then, $b^{T}\left(\sigma_{m}I\right)n=0$, so $\tau_{RSS}=b^{T}Sn$. If p is instead defined as a positive pressure, the decomposition is σ=S−pI. The distinction must be stated because writing σ=S+pI while calling p “pressure” reverses the usual sign convention [22,23,24]. Hydrostatic stress can still influence critical resolved shear stress (CRSS), phase stability, damage, diffusion, and other mechanisms; the correct conclusion is only that it supplies no direct Schmid shear on an orthogonal b−n system.

### 2.3. Normalization Is Part of the Model, Not a Cosmetic Choice

The quantity $b^{T}\sigma n$ has units of stress. A dimensionless global factor requires a reference stress, and different choices answer different questions. A factor normalized by a nominal tensile stress is convenient for a specified test. A factor normalized by the maximum principal stress may be meaningful in another context. A factor normalized by the von Mises equivalent stress compares the resolved shear projection with the magnitude of the deviatoric stress state. The generic expression is

(4) $$GSF=b^{T}\sigma n/\sigma_{ref}$$

Because $\sigma_{ref}$ is part of the definition, quantities carrying the same label “GSF” are not automatically comparable. The labels global, effective, and modified Schmid factor are not standardized across the literature. Every use should, therefore, cite the exact equation and report the stress tensor, coordinate frame, orientation convention, normalization, sign treatment, and crystallographic system. Normalization by a principal stress also requires care when that stress is zero, changes sign, or is not the relevant measure of deviatoric loading. Choi et al. [25], for example, combined Schmid and Taylor factors for a specific plane-strain roughening problem; that process-specific use should not be read as a universal GSF definition. Table 1 lists representative Schmid factor formulations and their interpretive scope.

## 3. A Rigorous Bound for Von Mises-Normalized GSF

The range of a global factor cannot be discussed without fixing its denominator. For the formulation used by Xia et al. [17] and further examined by Wang et al. [18], the reference stress is the von Mises equivalent stress:

(5) $$\sigma_{eq}=\bar{\sigma }=\sqrt{\frac{3}{2}s:s}$$

Choose an orthonormal basis in which $e_{1}=b$ and $e_{2}=n$. In this basis, $\tau_{RSS}=S_{12}$. Since

(6) $$s:s=s_{11}^{2}+s_{22}^{2}+s_{33}^{2}+2s_{12}^{2}+2s_{13}^{2}+2s_{23}^{2}$$

it follows that $s:s\geq 2s_{12}^{2}$, and, therefore,

(7) $$GSF=\left|\frac{\tau_{RSS}}{\bar{\sigma }}\right|=\left|\frac{s_{12}}{\bar{\sigma }}\right|\leq \frac{1}{\sqrt{3}}$$

**Figure 2:**
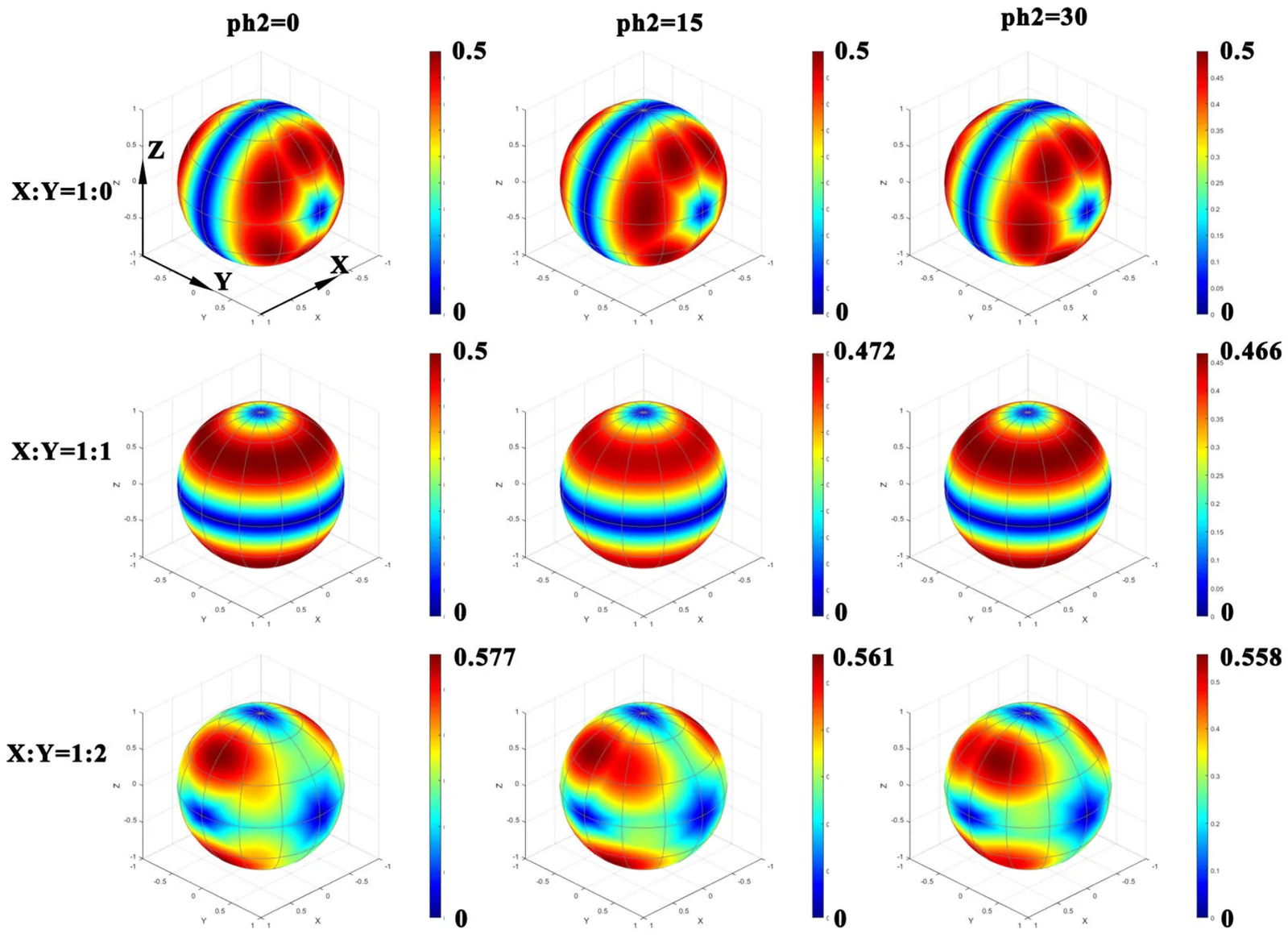
The variation in the GSF value range of basal slip with orientation under different stress states. Pixel points on the spherical surface indicate the spatial orientations of the c-axis of pure Mg grains at the sphere center, with colors denoting the corresponding GSF values.

Under the stated assumptions, the signed range is $[-1/\sqrt{3},\;1/\sqrt{3}],$ and the magnitude range is $\left[0,\;1/\sqrt{3}\right]$. These assumptions are essential: σ is a symmetric Cauchy stress tensor, b and n are orthogonal unit vectors, the denominator is the von Mises equivalent stress of the same non-hydrostatic stress state, and the numerator is the classical Schmid projection. Equality is attained for aligned pure shear. The value $1/\sqrt{3}$ is, therefore, a rigorous bound for this particular normalization, not for every quantity called a GSF and not for an effective factor containing additional normal-stress or energetic terms. Figure 2 shows the variation in GSF size and value range of basal slip with orientation under different stress states. It can be observed that the distribution of the GSF for basal slip within the magnesium alloy undergoes significant changes with variations in the stress state. Furthermore, the GSF distribution is also dependent on the specific value of the third Euler angle ph2. Based on these results, it can be inferred that under uniaxial tension in the X-direction, aligning the crystallographic c-axis of grains to approximately 45° with respect to the *Y*-axis of the sample coordinate system maximizes the GSF for basal slip. Such an orientation is expected to promote plasticity by enhancing the contribution of basal slip to the overall deformation [29,30].

Similarly, under equi-biaxial tension or compression in the X–Y plane, the GSF for basal slip approaches its maximum value of about 0.5 when the c-axis is oriented at roughly 45° to the *Z*-axis, making basal slip more readily activated to accommodate the imposed strain. For non-equi-biaxial loading in the X–Y plane, the GSF reaches its maximum only when the c-axis is simultaneously oriented at about 45° to both the X- and Z-axes; notably, this peak value can exceed the conventional upper bound of 0.5 and attain the theoretical limit of approximately 0.58. These findings offer theoretical guidance for tailoring crystallographic textures to improve ductility under various stress states.

Pure hydrostatic stress is a useful test. Here, $\tau_{RSS}=0$ and $\sigma_{eq}=0$, so the normalized ratio is mathematically undefined (0/0), not a GSF equal to zero. A robust implementation should return “not applicable: zero deviatoric stress” or *NaN* with an explanatory flag, while reporting the unnormalized resolved shear stress as zero. Adding a small numerical regularization without disclosure creates a value that has no invariant physical meaning. Table 2 shows the limiting stress states used to verify the calculation and its normalization. Under near-hydrostatic conditions where the deviatoric stress is very small, the external load can still generate some resolved shear stress on slip systems or twin shear directions, but the magnitude of this shear stress is governed by the level of deviatoric stress present. In materials with strong plastic anisotropy, the influence of even a small deviatoric component tends to be amplified compared with plastically isotropic materials. This is because the inherent anisotropy causes unequal straining in different directions under near-hydrostatic pressure, which, in turn, induces internal shear flow within the material [31,32,33].

## 4. What a Global Schmid Factor Can and Cannot Predict

### 4.1. A Projection Measure Is Not a Complete Activation Law

**Figure 3:**
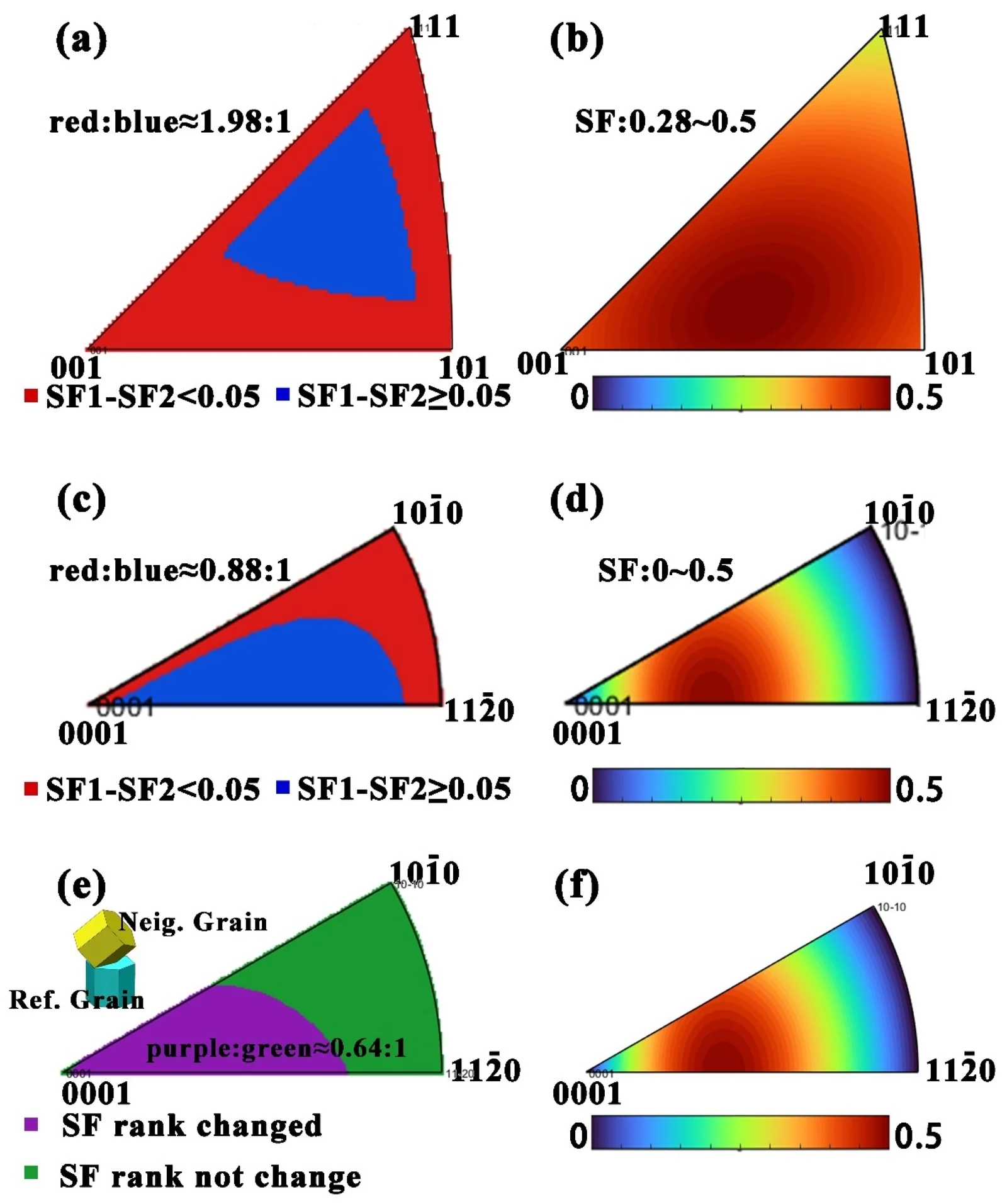
Orientation distributions (inverse pole figures) of grains where the difference between SF Rank 1 and SF Rank 2 for (**a**) {111}〈110〉 slip in FCC or (**c**) {0001}〈11–20〉 basal slip in HCP is less than 0.05 (red) or greater than 0.05 (blue) under loading in the X-direction. (**b**,**d**) Corresponding SF distributions for the active slip systems in (**b**) FCC and (**d**) HCP grains under X-direction loading. (**e**) For a reference grain with Euler angles (45°, 0°, 45°), the IPF map shows whether activation of basal slip in a neighboring grain (Neig. Grain)—through strain compatibility—can alter the SF Rank of basal slip variants in the reference grain (Ref. Grain). Orientations that induce a change in SF Rank are marked in purple; those that do not are marked in green. (**f**) SF of the neighboring grain with load along the *X*-axis.

A high factor means only that the prescribed stress state projects efficiently onto the selected system under the chosen normalization. The activation of slip α requires |$\tau_{RSS}^{\alpha }$| to reach the current CRSS $g^{\alpha }$; for a polar twin, the admissible sign and threshold must be defined for the selected variant. In a polycrystal, the relevant stress is the local grain stress, not necessarily the macroscopic applied stress. Temperature, strain rate, solute and precipitate state, grain size, hardening, and neighbor constraints modify either the resistance or the local driving force [34]. GSF is, therefore, a screening descriptor rather than a constitutive law. Consider the following case. If Schmid’s law were taken as the sole criterion, the onset of yielding in a given grain would depend merely on whether the applied load reaches the minimum stress required to activate any slip or twinning system within that grain. Consequently, macroscopic yielding of the polycrystalline aggregate would be governed primarily by this minimum stress and by the fraction of grains that have yielded. However, previous studies—including our own earlier work—have shown that such a constraint is overly restrictive [35,36,37,38,39]. In reality, some grains may yield prematurely under the accommodation of deformation from neighboring grains, even when their own intrinsic resistance to yielding is relatively high. This point will be revisited in Figure 3.

Across mechanisms with different resistances, a more informative but still conditional descriptor is the utilization ratio $U^{\alpha }=\tau_{RSS}^{\alpha }/g^{\alpha }$, with an absolute value used for bidirectional slip and a sign-aware form used for polar twinning. Even $U^{\alpha }$ does not predict the complete sequence of activity when latent hardening, non-Schmid effects, or grain interaction is important. For magnesium at room temperature, a non-basal system can have a larger GSF than basal <a> slip and, nevertheless, remain inactive because its CRSS is much higher [40]. It will be discussed later that the local stress effect between grains can ultimately be reflected in the magnitude of the resolved shear stress in the slip or shear direction.

### 4.2. Twinning Polarity and Stress-State Sensitivity

For slip, changing b to −b describes the opposite dislocation motion in the same system, so magnitude-based rankings are common. Deformation twins are polar. Reversing the shear sense generally corresponds to a different physical event, not merely a relabeled direction. Signed factors, therefore, carry information about whether a particular tensile or contraction twin variant is favored by the applied stress [10,11,16,41]. Discarding the sign before variant selection can erase the very asymmetry that the analysis is intended to reveal.

The sign of a twin-variant RSS or GSF is physically meaningful only after the twin shear direction and stress-sign convention are fixed. A negative value under a nominal stress state indicates that the nominal Schmid shear acts opposite to the selected polar twin sense. However, the magnitude of that negative nominal value cannot by itself be converted into a probability of later activation. Claims such as “−0.4 is less likely to be reversed than −0.05” require a quantified model or measurement of the local stress perturbation, variant resistance, and compatibility constraints. Neighbor slip or twinning may redistribute local stress and change the sign, but that possibility should be evaluated with local measurements or a crystal-plasticity calculation rather than inferred from the nominal GSF (calculated according to the macroscopic stress state) alone. For instance, in our previous in situ tensile study on AZ31 magnesium alloy, we observed that during uniaxial tension, several activated tensile twin variants exhibited Schmid factors below −0.1. Under biaxial tension, in contrast, only one tensile twin variant showed a generalized Schmid factor lower than −0.1 [41,42].

It should be noted that this discussion of positive and negative values is more pertinent to the nucleation stage of twins than to the growth stage because the stress levels and local strain compatibility required for twin nucleation are considerably lower—or less demanding—than those needed for subsequent twin growth.

### 4.3. Local Stress, Ebsd Post-Processing, and Crystal Plasticity

EBSD-based Schmid maps combine measured crystal orientations with an assumed stress state [43]. They, therefore, describe orientation favorability rather than the grain-scale stress tensor. Near grain boundaries, particles, twins, and free surfaces, local stresses can differ substantially from the nominal sample stress. Full-field crystal-plasticity methods and self-consistent polycrystal models account for stress redistribution and evolving resistance, although at higher parameter and computational cost [44,45]. High-resolution EBSD and diffraction-based methods provide complementary information on elastic strain and grain-resolved stress [14,46,47,48,49].

Disagreement between a nominal GSF ranking and an observed active system is therefore informative. It can indicate differences in CRSS, neighbor constraint, strain compatibility, or local stress concentration, rather than a failure of the tensor projection itself. Such comparisons are most useful when the assumed stress state and the evidence used to identify activity are reported independently.

For an FCC grain (e.g., pure aluminum) subjected to loading in the X-direction, the SFs of its 12 {111}〈110〉 slip systems vary with crystallographic orientation, as shown in Figure 3a. Ranking these 12 SF values, if the difference between Rank 1 and Rank 2 falls below 0.05, the two systems are considered nearly equally favored. Under such conditions, even minor fluctuations in the local stress field could readily reverse their ranking, manifesting macroscopically as non-Schmid behavior, that is, deformation that does not strictly follow Schmid’s law.

Figure 3b shows that under this stress state, the SF values for the {111}〈110〉 systems are generally high, with a minimum around 0.28. Combined with the red-to-blue orientation ratio of approximately 2:1 in Figure 3a—indicating that about 60% of orientations yield closely ranked SF values—this suggests a considerable likelihood of SF Rank alteration. This is precisely why many studies have reported frequent deviations from Schmid’s law in FCC metals.

Given the large number of slip variants (12) in FCC, which complicates the analysis of such rank fluctuations, the same approach was applied to HCP grains for a more tractable comparison. Figure 3c presents the difference between SF Rank 1 and Rank 2 for the three {0001}〈11–20〉 basal slip variants in HCP grains under X-direction loading, with the same 0.05 threshold used to mark orientations in red (difference <0.05) or blue (difference >0.05). The corresponding SF distribution for basal slip is shown in Figure 3d. Under this stress state, the basal SF spans a range of 0 to 0.5, with the lower bound noticeably below the 0.28 minimum seen in the FCC case. The red-to-blue orientation ratio is about 0.88:1, meaning that approximately 47% of orientations exhibit closely competing SF values, substantially lower than in FCC. This implies that non-Schmid effects for basal slip in HCP are comparatively less pronounced.

To further explore the role of neighboring grains, Figure 3e examines whether strain compatibility—specifically, slip activation in an adjacent grain—can alter the SF Rank of basal variants in a reference grain of fixed orientation ([45°, 0°, 45°]) under X-direction loading. The procedure is as follows: (1) Compute the SF values for the three basal variants in the reference grain. (2) Select an arbitrary orientation for a neighboring grain from the IPF space, compute its three basal SF values, and identify the variant with the largest absolute SF to represent the dominant slip contribution. (3) Treat the slip direction of this active variant as an effective loading direction imposed on the reference grain, with its SF magnitude serving as the load intensity ($\tau_{1}$, $\tau_{2}$, $\tau_{3}$, $\tau ={SF}_{n\_max}\cdot cos<n_{max},\;n_{i}>cos<b_{max},\;b_{i}>$, where $n_{max}$ and $n_{i}$ are the normal vectors of the basal planes of the neighboring grain and the reference grain, and $b_{max}$ and $b_{i}$ are the corresponding slip directions). Resolve this load onto the three basal slip directions of the reference grain to obtain additional shear components (signed, reflecting either promotion or suppression of each variant), then add these components to the original signed SF values of the reference grain, and take the absolute values to obtain updated SF values (${SF}_{1f}^{*}$, ${SF}_{2f}^{*}$, and ${SF}_{3f}^{*}$, where ${SF}_{1f}^{*}={SF}_{1f}+\tau_{1}$). (4) Rank the updated SF values and compare with the original ranking. If the ranking changes, mark the neighboring grain orientation in purple; otherwise, mark it in green.

The results in Figure 3e show that under the conditions examined, the ratio of neighboring orientations that cause an SF Rank change to those that do not is approximately 0.64:1, implying a roughly 39% probability that the activation hierarchy of basal slip in the reference grain is influenced by its neighbor. This probability is, of course, sensitive to the reference grain orientation, the number and actual orientation of neighboring grains, and the prevailing stress state. Figure 3e is intended primarily to illustrate the magnitude of this effect and the computational framework, rather than to offer a universal probability. In real deformation, such ranking fluctuations may occur between any two competing deformation modes, for example, between basal slip and tensile twinning.

SF and GSF are not intrinsic material properties and should not be presented as direct indicators of macroscopic yield strength, ductility, or remaining ductility. Their main materials science role is narrower but still useful. For a prescribed stress state, they identify which crystallographic systems are geometrically favored; distributions over many grains can reveal texture-controlled differences between loading directions and help screen candidate slip or twin variants. Such trends may contribute to an interpretation of anisotropy or mechanism competition, but the aggregate response also depends on CRSS distributions, elastic–plastic load sharing, hardening, grain morphology, boundary constraints, and damage evolution. The calculation of the SF or GSF is, in itself, a general procedure that does not depend on the specific material or lattice structure. As such, the associated formulations can be applied to any crystalline material that undergoes slip or twinning, such as Mg, Ti, Al, and steels, among others. However, the deformation characteristics of each material are by no means identical, and changes in external loading conditions can further alter the relative weighting of various factors that influence the deformation response. Therefore, when employing Schmid’s law to analyze deformation behavior, these contributing factors must be taken into due consideration in a balanced and context-specific manner.

For instance, at room temperature, the CRSS of the {0001}<11−20> basal slip system, the {10−10}<11−20> prismatic slip system, and the {10−11}<11−23> pyramidal <a+c> slip system in pure magnesium, which possesses an HCP lattice structure, differ considerably [50,51,52]. If the CRSS of the basal slip system is normalized to 1MPa, the CRSS of the prismatic slip system may be approximately 2MPa, and that of the pyramidal <a+c> slip system approximately 5MPa. Under an identical stress state, such as uniaxial tension in the X-direction, if the SFs for the three slip systems are 0.2, 0.3, and 0.45, respectively, the corresponding yield stresses required to activate each system are 5, 6.67, and 11.11 MPa. It is evident that even though the basal slip system exhibits the lowest SF, the actual external stress necessary to activate it remains the smallest; consequently, basal slip is still preferentially initiated. When the work-hardening effect is taken into account, once the plastic strain accumulated in the basal slip system reaches a certain level, such that its CRSS hardens to 1.33 MPa or above, the minimum external stress required to sustain basal slip increases to at least 6.67 MPa. Provided that grain rotation does not significantly alter the SF of the prismatic slip system, the external stress needed to continue basal slip becomes nearly equivalent to that required to initiate prismatic slip, and, thus, the prismatic slip system may also become activated. Similar phenomena can also be observed in titanium alloys. For example, pure titanium, which also has an HCP crystal structure at room temperature, exhibits the lowest CRSS for prismatic slip. Therefore, when the external loading and grain orientation conditions are analogous to those described above for pure magnesium, prismatic slip will be activated first. As work hardening brings the minimum external stresses required for activating prismatic and basal slip to comparable levels, both systems can be simultaneously activated. In the case of titanium alloys with a dual-phase structure, the slip systems that are preferentially activated in different phases may also induce strain compatibility across phase boundaries, thereby mutually promoting or suppressing their respective activations.

A valid conditional statement is, therefore, specific: if two grains experience the same known local stress form and magnitude, have the same relevant system resistance, and interactions can be neglected, the grain with the larger favorable RSS, or equivalently, the larger factor under a common normalization is expected to reach that activation threshold first. This makes SF/GSF a useful orientation-screening and hypothesis-forming tool. Once deformation changes orientation, local stress, or resistance, however, the factor must be recomputed. An initial value is a snapshot, not a measure of remaining ductility or an activation probability.

**Figure 4:**
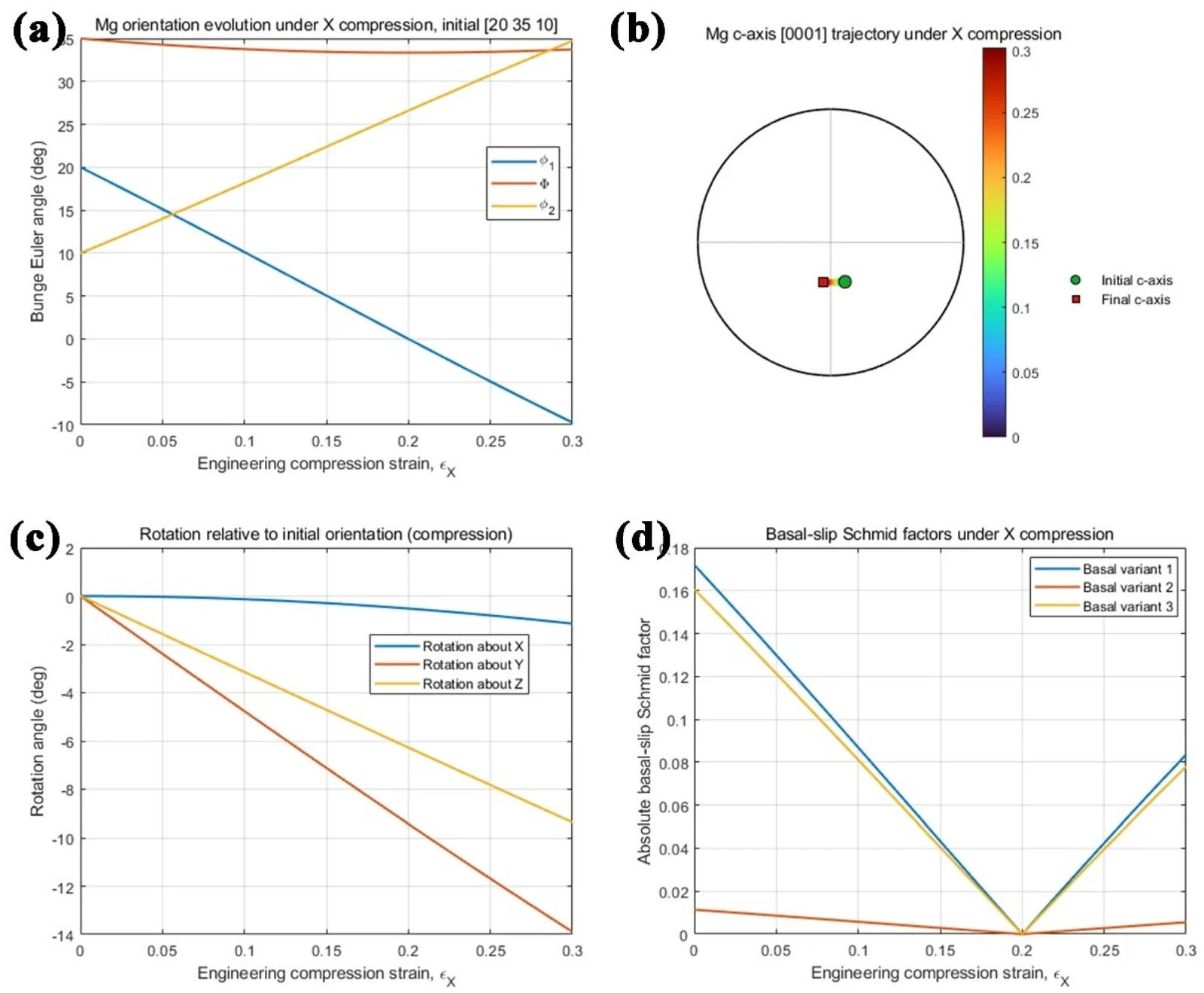
(**a**) Evolution of the three Bunge Euler angles with compressive strain in the X-direction for an HCP grain with an initial orientation of [20° 35° 10°]. (**b**) The orientation change in the {0001} pole figure; (**c**) rotation angles of the grain about the three sample axes as a function of compressive strain in the X-direction; and (**d**) SFs of the three basal slip variants versus compressive strain in the X-direction for the same HCP grain.

Take the case shown in Figure 4a,b: a magnesium grain with an initial orientation of [25° 35° 10°], compressed in the X-direction, undergoes noticeable orientation changes as strain accumulates. The rotations about the X-, Y-, and Z-axes differ considerably from one another (Figure 4c), and correspondingly, the Schmid factors of its three basal slip variants also evolve significantly (Figure 4d). Specifically, φ_1_ and φ_2_ shift by roughly 5° at a strain of 0.05, and the SF of basal variant 1 drops from about 0.17 to 0.13, a reduction of nearly 23.5%. This suggests that under certain loading conditions, a macroscopic compressive strain of around 5% can already produce substantial changes in both grain orientation and slip-system Schmid factors. In FCC materials, however, or when multiple slip mechanisms operate simultaneously to accommodate deformation, such effects tend to be much less pronounced.

## 5. Advice for Beginners

### 5.1. Common Conceptual Difficulties

The first difficulty is to regard SF as a property of a grain orientation alone. In fact, the value belongs to a relation among the crystal orientation, the selected deformation system, and the imposed stress state. Rotating either the crystal or the stress axes changes the projection. The second difficulty is to apply the uniaxial limit |m|≤0.5 to every normalized tensor measure. The third is to equate the largest factor with the active system, thereby omitting CRSS, twinning polarity, local stress, and hardening. These mistakes can produce numerically correct calculations with physically incorrect conclusions.

For an initial interpretation, SF or GSF should be read as a measure of directional loading efficiency: it quantifies how effectively the stated stress projects shear onto one specified plane–direction pair. It does not measure the amount of plastic strain, the probability of activation, or the strength of the material. This wording helps retain the physical meaning of the factor while preventing a geometric quantity from being assigned constitutive content that it does not contain.

When interpreted over a grain population, the factor can support several materials science inferences. A large fraction of grains with favorable basal-slip factors indicates that the texture makes basal slip geometrically accessible in the stated loading direction; it does not, by itself, establish a low yield stress. Differences between the SF distributions obtained when loading is applied in the rolling direction, transverse direction, and normal direction can identify the orientation contribution to plastic anisotropy. Signed twin-factor distributions can screen variants favored in tension or compression, while comparisons among slip families can suggest which mechanisms deserve further CRSS or activity analysis. In each case, the factor identifies a geometric tendency, and the material conclusion requires additional resistance or experimental information.

### 5.2. Learning Sequence

A useful learning sequence begins with the classical uniaxial geometry, where the loading direction, slip direction, and plane normal can be drawn, and the origin of 0.5 can be verified. The next step is to replace the loading direction with the full stress tensor and calculate $\tau_{RSS}=b^{T}\sigma n$ in a declared coordinate frame. Uniaxial tension, aligned pure shear, rigid rotation, and hydrostatic stress then provide compact checks of the implementation. Only after these checks should a normalized factor be used to rank systems.

The final step is to compare the geometric driving force with resistance and observation. For slip, $\left|\tau_{RSS}\right|$ may be compared with the relevant CRSS; for twinning, the sign and variant convention must also be retained. A lower-factor system that activates first is a productive counterexample rather than an inconvenience, because it directs attention to CRSS ratios, local stress redistribution, or strain compatibility. Learning research similarly supports the use of explicit prior conceptions, staged examples, and feedback when a compact formula is liable to be overgeneralized [53,54]. Additional calculation examples are provided in the Supplementary Materials to illustrate the influence of GSF on magnesium and other materials under various stress states. Figure S1 shows the stress tensors used, and Tables S1–S3 give the corresponding results for beginners to check their calculations. Table S4 offers a compact lesson plan for instructors.

### 5.3. Limitations of Schmid Law

Reliable use of SF or GSF requires the calculation to be separated from the subsequent physical inference. At a minimum, the crystallographic system, stress tensor, coordinate frame, orientation convention, numerator, normalization, and treatment of sign should be specified. For twinning, the polar shear direction must also be defined. For both learners and researchers, a maximum value or color map without these details cannot be reproduced and may not be comparable with results obtained using another convention.

Comparisons across samples or processing routes are meaningful only when the same definition and stress basis are retained. The distinction between nominal and local stress should be explicit, and differences in CRSS must be considered when competing mechanisms are ranked. Where possible, predicted slip or twin variants should be checked against slip traces, twin-boundary geometry, in situ diffraction, high-resolution EBSD, or crystal-plasticity calculations. A failed prediction should be reported rather than removed, because it helps identify whether local stress redistribution, path dependence, or non-Schmid behavior controls the response.

## 6. Outlook

### 6.1. From Nominal-Stress Maps to Local-Stress-Informed Descriptors

The most useful development is not another normalization, but a better estimate of the stress experienced by each grain. High-resolution EBSD, in situ diffraction, three-dimensional diffraction microscopy, digital image correlation, and crystal-plasticity simulations can be combined to examine whether rankings based on nominal stress survive local redistribution [46,47,55,56]. The remaining difficulty is to distinguish measurement uncertainty, model-dependent plastic stress and the evolution of grain orientation while retaining a transparent link to the crystallographic projection.

### 6.2. Path Dependence and Evolving System Resistance

A static GSF is inadequate once texture, lattice rotation, hardening, detwinning, or load reversal becomes significant. Future workflows should report $\tau_{RSS}^{\alpha }(t)$, orientation Q(t), and resistance $g^{\alpha }(t)$ along the loading path rather than only an initial scalar map. A path-dependent utilization ratio can be informative, but it must remain subordinate to a constitutive model when interactions and history effects dominate.

### 6.3. Uncertainty-Aware and Probabilistic Ranking

Orientation noise, uncertain boundary conditions, unknown local stress, and widely scattered CRSS values can change variant rankings. Propagating these uncertainties would replace a brittle statement such as “system A has the largest GSF” with a more defensible probability or confidence interval under stated assumptions. This direction is especially relevant when competing magnesium mechanisms have similar driving forces or when only two-dimensional EBSD data are available.

### 6.4. Standardization, Open Implementations, and Non-Schmid Extensions

The proliferation of global, effective, and modified factors makes standardized reporting and open reference implementations increasingly important. Shared unit tests based on uniaxial, pure-shear, hydrostatic, and rigid-rotation cases would expose sign and frame errors early. Non-Schmid or data-assisted extensions may be valuable for mechanisms that are not captured by a single shear projection, but additional terms should have a clear physical interpretation, independent calibration, and demonstrated predictive improvement over $\tau_{RSS}^{\alpha }/CRSS$. A more complicated scalar is not automatically a better model.

### 6.5. A Practical Research Agenda for Magnesium Alloys

For magnesium alloys, a productive agenda is to combine signed twin-variant projections, measured or simulated local stress, temperature- and rate-dependent CRSS, and in situ observations of activation. Studies should report both successful and failed predictions, distinguish nucleation from growth or propagation, and test whether conclusions transfer across textures and strain paths. The goal is to identify where the Schmid framework is sufficient, where local stress resolves apparent anomalies, and where genuinely non-Schmid physics is required.

## 7. Conclusions

The classical Schmid factor is the uniaxial specialization of $\tau_{RSS}=b^{T}\sigma n$. Its maximum magnitude of 0.5 is correct only for σ=1 with b·n=0. Under a nonzero deviatoric stress and von Mises normalization, $|\tau_{RSS}/\sigma_{eg}|\leq 1/\sqrt{3}$. This result is rigorous for that stated projection and normalization but is not a universal bound for all effective or modified factors.

The main interpretive correction is equally important: SF/GSF is an orientation-and-stress descriptor, not an intrinsic material property, complete activation law, or direct predictor of macroscopic strength or ductility. CRSS, twinning polarity, local stress, hardening, temperature, rate, strain path, and grain interaction must be considered. Negative twin GSF values retain sign information, but their magnitudes do not define activation probabilities without a model of local stress redistribution.

Accordingly, Schmid-based analysis should proceed from a clear geometric picture and a fully stated stress tensor, be checked against limiting stress states, and compare the calculated driving force with the relevant resistance. For first-time users, this progression provides a practical way to distinguish orientation favorability from material strength or mechanism activation. For research applications, further progress will depend on local-stress-informed, path-dependent, uncertainty-aware, and reproducible analyses, together with direct experimental tests of when a Schmid projection is sufficient and when additional physics is required.

## Figures and Tables

**Table 1:** Representative Schmid factor formulations and their interpretive scope.

Formulation	Core Expression or Idea	Best Used For	Main Caution
Classical SF	m=cosφcosλ	Uniaxial loading	0.5 bound is conditional under uniaxial stress
Tensor RSS	$\tau_{RSS}=b^{T}\sigma n$	Any specified Cauchy stress state	Stress activation criterion, not dimensionless
von Mises-normalized GSF	$GSF=b^{T}\sigma n/\sigma_{eq}$ $\sigma_{eq}=\sqrt{\frac{3}{2}s:s}$	Invariant comparison of non-hydrostatic stress states under one declared normalization	Undefined for a purely hydrostatic state because $\sigma_{eq}=0$
Effective Schmid factor	Adds work or energetic contributions associated with combined stress components [16]	Mechanism or variant selection in a defined process	The definition is formulation-specific and should not be treated as universal
Empirical formula	${SF}_{total}={SF}_{x}+0.5{SF}_{y}$ where ${SF}_{x},{SF}_{y}$ indicate the SF value when loaded along the X- and Y-axes	Applicable to sheet rolling or extrusion [26,27,28]	Similar to the GSF results, without normalization, not suitable for comparison with other stress states

**Table 2:** Limiting stress states used to verify the calculation of SF/GSF and its normalization.

Stress State	Resolved-Shear Implication	Normalized Result	Interpretive Use
Uniaxial tension	$\tau_{RSS}=\sigma \left(L\cdot b\right)\left(L\cdot n\right)=\sigma cos\varphi cos\lambda$	|m|≤0.5	Recovers the textbook formula and its conditional bound
Pure shear aligned with b and n	$\tau_{RSS}=\tau$	$\left|GSF\right|=1/\sqrt{3}\approx 0.577$	Demonstrates why 0.5 is not universal.
Biaxial or triaxial deviatoric loading	All tensor components may contribute through $b^{T}\sigma n$	Depends on stress ratios and orientation	There is no single geometric loading axis.
Hydrostatic pressure	$\tau_{RSS}=0$	$GSF=\left|\frac{\tau_{RSS}}{\bar{\sigma }}\right|$ undefined because $\bar{\sigma }=0$	Hydrostatic pressure does not directly drive Schmid shear

## Data Availability

No new data were created or analyzed in this study. Data sharing is not applicable to this article.

## Supplementary Materials

The following supporting information can be downloaded at: https://www.mdpi.com/article/10.3390/ma19173803/s1, Figure S1: Stress tensors used in the worked examples; arrow length indicates relative load magnitude; Table S1: Comparison of the two pure-Mg biaxial calculations.; Table S2: Signed RSS and GSF for all 12 FCC slip systems; rank is based on |GSF|; Table S3: Neighbor projection and updated basal-slip ranking in reference grain R; Table S4: Compact lesson plan.

## References

[1] Schmid E., Boas W. (1968). Plasticity of Crystals with Special Reference to Metals.

[2] Dieter G.E., Bacon D. (1976). Mechanical Metallurgy.

[3] Stokes R.J. (1969). Plasticity of crystals—With special reference to metals: E. Schmid and W. Boas. Pp. 368, Chapman and Hall Ltd., London, 1968 [Barnes and Noble, New York—U. S. Distributor]. Price: $8. 00. Mater. Res. Bull..

[4] Zhao Z., Wu W., Zhang B., Zhang Y., Zhang Z. (2026). Revealing strain distribution in metallic materials during in-situ SEM mechanical testing via HR-DIC: A review of instrumentation, methodology, and applications. Adv. Sci. Instrum..

[5] Shibano K., Suzuki T., Atugonza C., Alver N. (2026). Estimation of stress-strain relationship based on localized cracking phenomena for flexure and shear critical reinforced concrete beams under incremental cyclic loading using DIC and AE methods. Constr. Build. Mater..

[6] Lee M.-S., Ahn C.-W., Yoon J.W., Sohn S.S., Cho S., Jun T.-S., Park C.-S. (2026). In-situ DIC study of strain localisation and damage evolution in the B4C-reinforced aluminium metal matrix composite at high temperature. Mater. Sci. Eng. A.

[7] Xia D., Huang G., Liu S., Tang A., Gavras S., Huang Y., Hort N., Jiang B., Pan F. (2019). Microscopic deformation compatibility during biaxial tension in AZ31 Mg alloy rolled sheet at room temperature. Mater. Sci. Eng. A.

[8] Sun J., Jin L., Dong J., Ding W., Luo A.A. (2016). Microscopic deformation compatibility during monotonic loading in a Mg-Gd-Y alloy. Mater. Charact..

[9] Zhang S., Godfrey A., Zhang C., Liu W., Juul Jensen D. (2020). Surface patterning for combined digital image correlation and electron backscatter diffraction in-situ deformation experiments. Mater. Charact..

[10] Christian J.W., Mahajan S. (1995). Deformation twinning. Prog. Mater. Sci..

[11] Yoo M.H. (1981). Slip, twinning, and fracture in hexagonal close-packed metals. Metall. Trans. A.

[12] Nan X.-L., Wang H.-Y., Zhang L., Li J.-B., Jiang Q.-C. (2012). Calculation of Schmid factors in magnesium: Analysis of deformation behaviors. Scr. Mater..

[13] Nan X.-L., Wang H.-Y., Wu Z.-Q., Xue E.-S., Zhang L., Jiang Q.-C. (2013). Effect of c/a axial ratio on Schmid factors in hexagonal close-packed metals. Scr. Mater..

[14] Agnew S.R., Duygulu Ö. (2005). Plastic anisotropy and the role of non-basal slip in magnesium alloy AZ31B. Int. J. Plast..

[15] Lou X.Y., Li M., Boger R.K., Agnew S.R., Wagoner R.H. (2007). Hardening evolution of AZ31B Mg sheet. Int. J. Plast..

[16] Chen S.-F., Song H.-W., Zhang S.-H., Cheng M., Zheng C., Lee M.-G. (2019). An effective Schmid factor in consideration of combined normal and shear stresses for slip/twin variant selection of Mg-3Al-1Zn alloy. Scr. Mater..

[17] Xia D., Chen X., Huang G., Jiang B., Tang A., Yang H., Gavras S., Huang Y., Hort N., Pan F. (2019). Calculation of Schmid factor in Mg alloys: Influence of stress state. Scr. Mater..

[18] Wang W., Chen X., Hong Y., Huang G., Chen X., Li J., Zheng K., Jiang B., Pan F. (2025). Modified Schmid factor analysis and calculation of magnesium crystals under three-dimensional stress states. J. Mater. Res. Technol..

[19] Zhang Y., Zhou Y., Liu S., Xin R. (2026). A unified composite Schmid factor approach to deformation twin pair selection in pure titanium. J. Alloys Compd..

[20] Gere J.M., Timoshenko S.P. (1997). Mechanics of Materials.

[21] Gere J.M. (2012). Mechanics of Materials.

[22] Shunyingve J.I., Mei Y., Marmysh D. (2024). Mechanics of Materials.

[23] Öchsner A. (2014). Continuum Mechanics of Plasticity.

[24] Ohashi T., Kuroda M. (2013). Mechanics of Plasticity at the Micron Scale. J. Jpn. Soc. Technol. Plast..

[25] Choi Y.S., Piehler H.R., Rollett A.D. (2004). Formation of mesoscale roughening in 6022-T4 Al sheets deformed in plane-strain tension. Metall. Mater. Trans. A.

[26] Luo J.R., Godfrey A., Liu W., Liu Q. (2012). Twinning behavior of a strongly basal textured AZ31 Mg alloy during warm rolling. Acta Mater..

[27] Barnett M.R., Keshavarz Z., Nave M.D. (2005). Microstructural features of rolled Mg-3Al-1Zn. Metall. Mater. Trans. A.

[28] Guo C. (2016). Strain Accommodation Effect Between Deformation Twins in Magnesium Alloys and Their Variant Selection Mechanism. Ph.D. Thesis.

[29] Tan C., Zhang Y., Wang B., Xin R. (2025). Quantifying the role of texture in the compression anisotropy of AZ31 Mg alloy via crystal plasticity finite element modeling. J. Alloys Compd..

[30] Tan C., Zhang Y., Ren W., Xin R., Chen X. (2026). Unraveling texture-dependent fracture in magnesium alloy: A combined EBSD and crystal plasticity study. J. Alloys Compd..

[31] Li Z., Wu G., Liu Y., Yu J., Zhang Z. (2023). The effect of hydrostatic pressure on grain refinement and deformation behaviors of Mg–Gd–Y–Zn–Zr alloy under complex shear stress. J. Mater. Res. Technol..

[32] Mears D.R., Pae K.D., Sauer J.A. (1969). Effects of Hydrostatic Pressure on the Mechanical Behavior of Polyethylene and Polypropylene. J. Appl. Phys..

[33] (2021). New Findings from Chinese Academy of Sciences in Metallurgy Provides New Insights (Effect of Hydrostatic Pressure on Lpso Kinking and Microstructure Evolution of Mg-11gd-4y-2zn-0.5zr Alloy). Min. Miner..

[34] Kocks U.F., Mecking H. (2003). Physics and phenomenology of strain hardening: The FCC case. Prog. Mater. Sci..

[35] Xia D., Wu B., Xu L., Yang G., Huang G., Gupta M., Yang H., Zhao C., Wang L. (2024). A new progressive polycrystalline yield criterion for magnesium alloys. Comput. Mater. Sci..

[36] Pelleg J. (2013). Mechanical Properties of Materials.

[37] Gay D., Gambelin J. (2008). Mechanical Properties of Materials (Appendix B). Modeling and Dimensioning of Structures: A Practical Approach.

[38] Zheng Z., Xie C., Chen J., Huang Z. (2023). A crystal plasticity model of low cycle fatigue damage considering dislocation density, stress triaxiality and Lode parameter. Int. J. Fatigue.

[39] Yang S., Guo X., Ma C., Shen L., Zhao L., Zhu W. (2025). Anisotropic Mechanical Behavior in an Extruded AZ31 Magnesium Alloy: Experimental and Crystal Plasticity Modeling. Acta Metall. Sin. (Engl. Lett.).

[40] Chen W., Li X., He W., Jiang B., Pan F. (2025). Achieving excellent ductility with increased Schmid Factor but decreased direct strain contribution of basal slip in an intergranular misorientation tailored magnesium alloy. Acta Mater..

[41] Li T., He Q., Liu S., Chen W., He L., Luo J., Yin D., Zheng J., Jiang B., Pan F. (2025). The high dependence of non-Schmid tension twinning behavior on intergranular misorientation in magnesium under hard-orientation loading. Scr. Mater..

[42] Xia D., Huang G., Deng Q., Jiang B., Liu S., Pan F. (2018). Influence of stress state on microstructure evolution of AZ31 Mg alloy rolled sheet during deformation at room temperature. Mater. Sci. Eng. A.

[43] Wright S.I., Nowell M.M., Field D.P. (2011). A Review of Strain Analysis Using Electron Backscatter Diffraction. Microsc. Microanal..

[44] Roters F., Eisenlohr P., Hantcherli L., Tjahjanto D.D., Bieler T.R., Raabe D. (2010). Overview of constitutive laws, kinematics, homogenization and multiscale methods in crystal plasticity finite-element modeling: Theory, experiments, applications. Acta Mater..

[45] Lebensohn R.A., Tomé C.N. (1993). A self-consistent anisotropic approach for the simulation of plastic deformation and texture development of polycrystals: Application to zirconium alloys. Acta Metall. Mater..

[46] Wilkinson A.J., Meaden G., Dingley D.J. (2006). High-resolution elastic strain measurement from electron backscatter diffraction patterns: New levels of sensitivity. Ultramicroscopy.

[47] Britton T.B., Wilkinson A.J. (2012). High resolution electron backscatter diffraction measurements of elastic strain variations in the presence of larger lattice rotations. Ultramicroscopy.

[48] Penttilä J., Nyyssönen T., Kuokkala V.-T., Raami L., Peura P. (2026). Schmid factor aided slip pattern analysis; A method for determination of orientations of FCC crystals from slip traces. Mater. Charact..

[49] Ji Y., Suo H., Zhang Z. (2025). Connection between stacking fault energy and rolling-texture type in Ni and Ni-W alloys using Schmid factor as bridge. Mater. Charact..

[50] Mo Y., Su J., He S., Yang J., Xu H., Teng J., Zhang H., Jiang F. (2026). Insights into the texture-dependent strain hardening mechanisms of conform-processed AZ31 Mg alloys: Experimental investigations and VPSC modeling. J. Magnes. Alloys.

[51] Chen X., Lin J., Wang Z. (2026). Research on Deformation Mechanism of Rolled AZ31B Magnesium Alloy during Tension by VPSC Model Computational Simulation. Comput. Mater. Contin..

[52] Xia D., Zhang J., Chen X., Huang G., Jiang B., Tang A., Gavras S., Huang Y., Hort N., Pan F. (2020). Effect of biaxial compressive stress state on the microstructure evolution and deformation compatibility of rolled sheet Mg alloy AZ31 at room temperature. Mater. Sci. Eng. A.

[53] Ambrose S.A., Bridges M.W., Dipietro M., Lovett M.C., Norman M.K. (2012). How Learning Works: Seven Research-Based Principles for Smart Teaching. J. Chiropr. Educ..

[54] Illas Limson S.L. (2025). How People Learn II: Learners, Contexts, and Cultures. Acad. Manag. Learn. Educ..

[55] Roters F., Diehl M., Shanthraj P., Eisenlohr P., Reuber C., Wong S.L., Maiti T., Ebrahimi A., Hochrainer T., Fabritius H.O. (2019). DAMASK—The Düsseldorf Advanced Material Simulation Kit for modeling multi-physics crystal plasticity, thermal, and damage phenomena from the single crystal up to the component scale. Comput. Mater. Sci..

[56] Ralph B. (2005). *Three-Dimensional X-Ray Diffraction Microscopy (Mapping Polycrystals and Their Dynamics)*[Book Review]. Mater. Charact..

