# Supplementary material for: From the Classical Schmid Factor to Tensor-Based Global Measures: A Critical Review and Practical Advice for Beginners
Source: Materials (Basel). 2026 Sep 7;19(17):3803. doi: 10.3390/ma19173803 (PMC13567086; doi:10.3390/ma19173803)
Supplement: Supplementary file 1 [file materials-19-03803-s001.zip › materials-4509924-supplementary.pdf]

SUPPLEMENTARY MATERIAL: Worked Global Schmid Factor Calculations for Biaxial Mg, Tension-Compression Al, and Two-Grain Mg Coordination

1. Calculation Framework

The examples use the tensor projection and von Mises normalization adopted in the main manuscript. The stress tensor, slip direction b, and plane normal n are all expressed in the sample X-Y-Z frame. Both b and n are unit vectors and $b \cdot n = 0$. The signed resolved shear stress and dimensionless Global Schmid factor are

$$\tau_{RSS} = b^T \sigma n \tag{S1}$$

$$S = \sigma - \sigma_m I; \ \sigma_{eq} = \sqrt{\frac{3}{2} s : s} \tag{S2}$$

$$GSF = b^T \sigma n / \sigma_{eq}, \ \sigma_{eq} > 0 \tag{S3}$$

For ordinary slip, opposite directions $\pm b$ belong to the same slip system, so magnitudes are used for ranking after the signed calculation. GSF describes geometric loading efficiency under the prescribed stress; activation additionally requires the relevant CRSS and, in a polycrystal, the local grain stress.

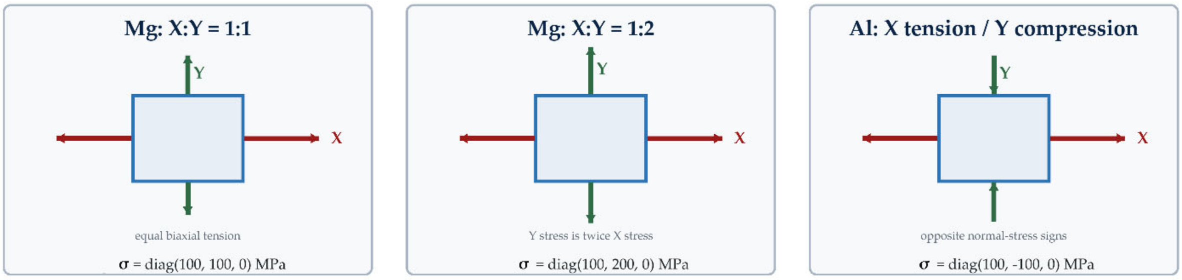

Figure S1. Stress tensors used in the worked examples; arrow length indicates relative load magnitude.

Coordinate and Orientation Convention

If crystallographic vectors are supplied in the crystal frame, they must first be transformed by the declared crystal-to-sample orientation matrix g: $b = gb$ and $n = gn$. To keep the arithmetic auditable, the Mg examples below provide sample-frame vectors directly, while the Al example sets $g = I$ so that [100], [010], and [001] coincide with X, Y, and Z.

Case	Stress state	Systems evaluated	Main point
Pure Mg	X-Y biaxial tension	One transparent basal variant	Stress ratio changes GSF and its bound.
Pure Al	X tension / Y compression	12 {111}<110> systems	Rank all FCC systems with one tensor.
Two pure Mg grains	X uniaxial tension	Three basal variants per grain	Neighbor compatibility can reverse rank.

*Interpretive boundary. The two-grain update on page 4 is the screening construction described in the manuscript: it represents an additional compatibility projection, not a solved local Cauchy stress from crystal plasticity.

2. Pure Mg under X-Y Biaxial Tension

For $\sigma = diag(1, r, 0)$, where $r = \sigma_Y/\sigma_X > 0$, the von Mises equivalent stress and the basal-system projection are

$$\sigma_{eq} = \sqrt{\frac{r^2+1+(1+r)^2}{2}} \quad \text{(S4)}$$

$$GSF = b^T\sigma n/\sigma_{eq} \quad \text{(S5)}$$

The following vectors represent valid basal slip systems in pure Mg because n is the basal-plane normal (the c-axis), b lies in that plane, and $b \cdot n = 0$. Reversing b changes only the sign for ordinary slip.

2.1 Equal Biaxial Tension, X:Y = 1:1

Let σ_0= 100 MPa, $n = (1/\sqrt{2}, 0, 1/\sqrt{2})$, and $b = (1/\sqrt{2}, 0, -1/\sqrt{2})$. The c-axis is 45° from Z in the X-Z plane. Then

$$\sigma = diag(100, 100, 0) MPa, \; \sigma_{eq}=100 MPa \quad \text{(S6)}$$

$$\tau_{RSS} = b^T\sigma n = 50\ MPa \quad \text{(S7)}$$

$$GSF = 50/100 = 0.500 \quad \text{(S8)}$$

2.2 Unequal Biaxial Tension, X:Y = 1:2

Now use $\sigma = diag(100, 200, 0)$ MPa and select $n = (0, 1/\sqrt{2},\ 1/\sqrt{2})$, $b = (0, 1/\sqrt{2},\ -1/\sqrt{2})$. This basal variant bisects the Y-Z principal directions associated with the largest principal-stress difference.

$$s = diag(0, 100, -100)\ MPa; \; \sigma_{eq} = 173.205\ MPa \quad \text{(S9)}$$

$$\tau_{RSS} = 200(1/2) = 100\ MPa \quad \text{(S10)}$$

$$GSF = 100/173.205 = 0.57735 = 1/\sqrt{3} \quad \text{(S11)}$$

Table S1. Comparison of the two pure-Mg biaxial calculations.

X:Y	Representative c-axis n	Representative basal b	τ_{RSS} (MPa)	GSF
1:1	(0.7071, 0, 0.7071)	(0.7071, 0, -0.7071)	50.000	0.5000
1:2	(0, 0.7071, 0.7071)	(0, 0.7071, -0.7071)	100.000	0.5774

Result: The value 0.5 is recovered for equal biaxial tension in the selected orientation, whereas unequal biaxial tension can reach $1/\sqrt{3}$. A GSF above 0.5 is therefore not an error when the full tensor and von Mises normalization are used. Across an orientation population, the active basal variant and its value must be recalculated for each grain

Material interpretation remains conditional: pure Mg basal slip usually has a lower room-temperature CRSS than prismatic or pyramidal slip, but GSF alone does not supply CRSS, hardening, or grain-neighbor constraint.

3. Pure Al under X Tension and Y Compression

Take $\sigma = diag(100, -100, 0)$ MPa and $g = I$ for an FCC Al crystal. The stress is already deviatoric, so σ_{eq}= 173.205 MPa. Each of the 12 {111}<110> systems is evaluated with Equation (S1). One detailed example is

$n\_(111) = (1,1,1)/\sqrt{3}; b\_[1-10] = (1,-1,0)/\sqrt{2}$ (S12)

τ_{RSS} *= 81.650 MPa; GSF = 0.47140* (S13)

Table S2. Signed RSS and GSF for all 12 FCC slip systems; rank is based on $|GSF|$.

Plane	Direction	τ_{RSS} (MPa)	GSF	Magnitude rank
(111)	[1 −1 0]	+81.650	+0.4714	1
(1 −1 1)	[1 1 0]	+81.650	+0.4714	1
(−1 1 1)	[1 1 0]	-81.650	-0.4714	1
(1 1 −1)	[1 −1 0]	+81.650	+0.4714	1
(111)	[0 1 −1]	-40.825	-0.2357	2
(111)	[1 0 −1]	+40.825	+0.2357	2
(1 −1 1)	[0 1 1]	+40.825	+0.2357	2
(1 −1 1)	[1 0 −1]	+40.825	+0.2357	2
(−1 1 1)	[0 1 −1]	-40.825	-0.2357	2
(−1 1 1)	[1 0 1]	-40.825	-0.2357	2
(1 1 −1)	[0 1 1]	-40.825	-0.2357	2
(1 1 −1)	[1 0 1]	+40.825	+0.2357	2

Result: Four systems tie at $|GSF|$ = 0.4714 and eight tie at $|GSF|$ = 0.2357. Because opposite slip directions are equivalent, the sign identifies shear sense but not a different ordinary slip system. The four-way tie also shows why a small local stress perturbation can change the observed slip-system ranking in FCC Al.

This result belongs to $g = I$. Rotating the Al grain changes b and n in the sample frame and therefore changes every entry in Table S2, although the calculation procedure is unchanged.

4. Coordination between Two Pure-Mg Grains under X Tension

Let $\sigma = diag(100, 0, 0)$ MPa; then σ_{eq}= 100 MPa and the signed uniaxial SF equals the signed GSF. To make the neighbor calculation transparent, the reference grain R is defined directly by its basal normal and three in-plane directions:

$n_r = (0.7071,0,0.7071)$ (S14)

$b_{r1} = (0.7071,0,-0.7071)$ (S15)

$b_{r2} = (-0.3536,0.8660,0.3536);\ b_{r3} = (-0.3536,-0.8660,0.3536)$ (S16)

The original signed SF values are $(0.500,-0.250,-0.250)$, so variant R1 initially ranks first. For the neighbor grain N, choose the dominant basal system

$b_N = (-0.7071,-0.7071,0);\ n_N = (-0.7071,0.7071,0)$ (S17)

Its signed SF is $m_N = 0.5$. Following the manuscript's screening construction, use $I_N = |m_N|$ as the neighbor load intensity, project b_N onto each reference direction, and update the signed reference values:

$c_i = b_{ri} \cdot b_N;\ \Delta\, m_i = I_N\, c_i;\ m_i^* = |m_i^0 + \Delta\, m_i|$ (S18)

Table S3. Neighbor projection and updated basal-slip ranking in reference grain R.

Variant	m_i^0	$c_i = b_{ri} \cdot b_N$	$\Delta\, m_i$	Signed sum	m_i^* / rank
R1	+0.5000	-0.5000	-0.2500	+0.2500	0.2500 / 2
R2	-0.2500	-0.3624	-0.1812	-0.4312	0.4312 / 1
R3	-0.2500	+0.8624	+0.4312	+0.1812	0.1812 / 3

Coordination effect: The neighbor contribution suppresses R1 from 0.500 to 0.250 and raises the magnitude of R2 from 0.250 to 0.431; the preferred reference-grain variant therefore changes from R1 to R2. This demonstrates how compatibility may promote one variant and suppress another even when the nominal X stress is unchanged.

Caution: Equation (S18) is an illustrative ranking update taken from the manuscript's neighbor-effect framework. A quantitative local-stress prediction requires grain geometry, boundary conditions, constitutive parameters, and a crystal-plasticity or experimentally constrained stress solution.

5. Simple Undergraduate Teaching Design

Format: one 50-minute class for second- or third-year materials-science students who know stress tensors, dot products, and basic slip crystallography. The lesson uses the three worked cases above to move from calculation to interpretation.

Learning Outcomes

By the end of class, students can
1. Calculate τ_{RSS}, σ_{eq}, and GSF in one declared coordinate frame.
2. Explain why 0.5 is not a universal GSF bound under multiaxial loading.
3. Rank FCC slip systems and distinguish geometric ranking from activation.
4. Explain qualitatively how a neighbor may promote or suppress a Mg basal variant.

Fifty-Minute Sequence

Table S4. Compact lesson plan.

Time	Teaching move	Student task	Evidence
0-5 min	Pose: Is 0.5 always the maximum?	Vote and justify in one sentence.	Prior conception is visible.
5-13 min	Derive Equations S1-S3.	Label sigma, b, n, frame, and sign.	Correct inputs are identified.
13-23 min	Model the two Mg biaxial cases.	Calculate σ_{eq} and compare 0.500 with 0.577.	Student states why >0.5 is valid.
23-34 min	Assign FCC systems in small groups.	Calculate one Al row, then assemble the 12-system ranking.	Four systems tie for rank 1.
34-44 min	Introduce neighbor compatibility.	Complete one row of Table S3 and predict the new rank.	R2 replaces R1 as rank 1.
44-50 min	Separate projection from activation.	Complete the exit ticket individually.	Instructor identifies needed reteaching.

Student Prompts and Answer Checks

Prompt	Expected check
Why does Mg X:Y = 1:2 give 0.577 rather than 0.5?	The denominator is von Mises stress and the selected basal system resolves the maximum principal-stress difference.
Which Al systems are most favored for $g = I$?	The four rows with $\|GSF\|$ = 0.4714; sign alone does not create a new ordinary slip system.
What changed the Mg reference-grain ranking?	The signed neighbor projection Δm_i promoted R2 and suppressed R1.

Prompt	Expected check
Does rank 1 prove activation?	No. CRSS, local stress, hardening, and experimental evidence are also needed.

Exit ticket: Write the GSF equation, name one assumption needed for comparison, and give one reason that the largest GSF may not identify the observed active system.

Method note: Equations S1-S3, the von Mises-normalized bound, the FCC ranking logic, and the neighbor-grain coordination construction follow the original manuscript. The explicit vectors and numerical stress magnitudes in this supplement are selected to make each calculation reproducible in a short teaching format.
